# Insomnia and psychological distress in lung cancer

**DOI:** 10.1192/j.eurpsy.2025.1770

**Published:** 2025-08-26

**Authors:** M. Abdelkefi, I. Gassara, R. Jbir, S. Omri, B. Abderrahmane, R. Khemakhem, R. Feki, N. Smaoui, J. Ben Thabet, M. Maalej, M. Maalej, S. Kammoun, N. Charfi, L. Zouari

**Affiliations:** 1 Psychiatry C department; 2 Department of pneumology, Hedi Chaker university hospital, Sfax, Tunisia

## Abstract

**Introduction:**

The high symptom burden of lung cancer, coupled with the emotional strain of diagnosis and treatment, often leads to disrupted sleep patterns and heightened levels of anxiety and depression.

**Objectives:**

The aim of this study was to assess the prevalence and severity of insomnia in patients with primary bronchopulmonary cancers, and to explore its relationship with anxiety and depression.

**Methods:**

This was a cross-sectional, descriptive and analytical study conducted among patients followed up for bronchopulmonary cancer at the palliative care unit in the pneumology and allergology department of the Hedi Chaker University Hospital in Sfax. The questionnaire used included patients’ sociodemographic characteristics, clinical and treatment data. Sleep disturbance was assessed using the insomnia severity index (ISI) and psychological distress using the Hospital Anxiety and Depression Scale (HADS).

**Results:**

A total of 49 patients participated, with a mean age of 61,8 years, the majority being male (85,7%). The disease duration was less than one year in 61,2% of cases, and 59,2% of patients had stage IV lung cancer, with tumor progression observed in 32,7%.

Mild to moderate insomnia affected 49% of the patients.

The mean anxiety score was 7,08 (SD = 3,6), with a prevalence of anxiety observed in 4,1% of patients. The mean depression score was 6,92 (SD = 3,4), and 34,7% of patients exhibited signs of depression.

A significant association was found between insomnia and depression (p=0,005), but no significant relationship was observed between insomnia and anxiety (p=0,14).

**Conclusions:**

The relationship between sleep disturbance and psychological distress highlights the need for comprehensive management that addresses both physical symptoms and mental health in this population. Early identification and intervention for insomnia and depression in lung cancer patients may enhance their overall well-being and quality of life.

**Disclosure of Interest:**

None Declared

